# Automatic Detection of Diabetic Retinopathy in Retinal Fundus Photographs Based on Deep Learning Algorithm

**DOI:** 10.1167/tvst.8.6.4

**Published:** 2019-11-12

**Authors:** Feng Li, Zheng Liu, Hua Chen, Minshan Jiang, Xuedian Zhang, Zhizheng Wu

**Affiliations:** 1 School of Optical-Electrical and Computer Engineering, University of Shanghai for Science and Technology, Shanghai 200093, China; 2 Department of Precision Mechanical Engineering, Shanghai University, Shanghai 200072, China

**Keywords:** diabetic retinopathy, retinal fundus photographs, deep transfer learning, Inception-v3 network

## Abstract

**Purpose:**

To achieve automatic diabetic retinopathy (DR) detection in retinal fundus photographs through the use of a deep transfer learning approach using the Inception-v3 network.

**Methods:**

A total of 19,233 eye fundus color numerical images were retrospectively obtained from 5278 adult patients presenting for DR screening. The 8816 images passed image-quality review and were graded as no apparent DR (1374 images), mild nonproliferative DR (NPDR) (2152 images), moderate NPDR (2370 images), severe NPDR (1984 images), and proliferative DR (PDR) (936 images) by eight retinal experts according to the International Clinical Diabetic Retinopathy severity scale. After image preprocessing, 7935 DR images were selected from the above categories as a training dataset, while the rest of the images were used as validation dataset. We introduced a 10-fold cross-validation strategy to assess and optimize our model. We also selected the publicly independent Messidor-2 dataset to test the performance of our model. For discrimination between no referral (no apparent DR and mild NPDR) and referral (moderate NPDR, severe NPDR, and PDR), we also computed prediction accuracy, sensitivity, specificity, area under the receiver operating characteristic curve (AUC), and κ value.

**Results:**

The proposed approach achieved a high classification accuracy of 93.49% (95% confidence interval [CI], 93.13%–93.85%), with a 96.93% sensitivity (95% CI, 96.35%–97.51%) and a 93.45% specificity (95% CI, 93.12%–93.79%), while the AUC was up to 0.9905 (95% CI, 0.9887–0.9923) on the independent test dataset. The κ value of our best model was 0.919, while the three experts had κ values of 0.906, 0.931, and 0.914, independently.

**Conclusions:**

This approach could automatically detect DR with excellent sensitivity, accuracy, and specificity and could aid in making a referral recommendation for further evaluation and treatment with high reliability.

**Translational Relevance:**

This approach has great value in early DR screening using retinal fundus photographs.

## Introduction

The main complication of diabetes is diabetic retinopathy (DR), a retinal vascular disease, which can lead to visual impairment or permanent blindness if not discovered in its initial stages.^1^,^2^ Early diagnostic and regular eye examinations^3^–^5^ could prevent vision loss and blindness and played a crucial role in optimal treatment of DR. Retinal screening as a conventional and efficient solution is performed for the early diagnosis of DR using retinal fundus photographs obtained with a mydriatic or nonmydriatic camera by experienced optometrists or specialized, highly trained eye technicians.^6^,^7^ However, traditional manual DR screening remains challenging and is subject to substantial inter- and intraobserver variability, even among experienced ophthalmologists, which can result in making inconsistent interpretation, delaying accurate diagnosis and creating a drain on health-care resources.^8^,^9^ Hence, the importance of automated detection for DR on screened color fundus images has been recognized.

In recent years, many previous studies have mentioned state-of-the art applications of deep learning algorithms^10^–^13^ in automated detection of retinal diseases from a large number of fundus images. Mookiah et al.^14^ applied discrete wavelet transform and stationary wavelet transform coefficients to extract features from retinal fundus images, and the optimum features were selected for distinguishing normal from DR by means of top-rank and AdaBoost machine learning methods. Akram et al.^15^ presented a hybrid classifier integrating a Gaussian mixture model with a classifier based on the m-Mediods to detect dark and bright regions for recognizing DR-related lesions. Haloi et al.^16^ developed a pixel-level microaneurysm classification approach based on deep learning for early DR screening in color fundus images, which was barely affected by luminance, contrast changes, and artifacts. Abràmoff et al.^17^ investigated performance of a hybrid deep learning model with multiple convolutional neural networks (CNNs) trained as lesion detectors on the free Messidor-2 dataset to automatically detect retinal lesions and normal anatomy. Gulshan et al.^18^ applied a transfer learning method to detect referable DR and gradable images in retinal color fundus images, generating high sensitivity and specificity. Zeng et al.^19^ utilized a Siamese-like CNN based on binocular retinal fundus images trained with a transfer learning technique for automated DR detection, which achieved high performance.

Although the abovementioned studies released superior performance in controlled experimental circumstances, there were some limitations. These classification approaches introduced above^14^,^15^ relied on handcrafted features, which required abundant professional knowledge and resulted in a time-consuming process, poor generalization, and even unfeasibility in large datasets. These deep learning methods^16^–^18^ utilized the raw images to train a blank CNN, which required an extremely large labeled training data and could be a very time-consuming task. The transfer learning approach^19^ had no further optimization, leading to a decline of predictive power. Moreover, the constructions of these models with data-driven feature quantifiers on inadequate amounts of data led to overfitting, which had a negative impact on performance during testing. In addition, some studies made only binary classification. In real clinical circumstances, patients often suffered from a variety of retinal disorders, which employing the binary classification difficult in reality. As such, multicategorical classification emphasizing detecting various ocular disorders was more suitable for the actual clinical setting. However, the implementation of multiclass classification had significant challenges.

In order to address the above limitations and maximize the clinical utility of automated detection, in this study the deep transfer learning method using the Inception-v3 network was explored for automatically categorizing any DR present in retinal fundus photographs as no apparent DR, mild/moderate/severe nonproliferative DR (NPDR), and proliferative DR (PDR) to assign the level of DR progression. The proposed approach, with its high accuracy, high sensitivity, and high specificity, could assist in making automated screening for early DR based on retinal fundus photographs and potentially alleviate the demand for the resource-intensive manual analysis of retinal fundus photographs from diverse clinical circumstances so that high-risk patients could be effectively referred for further evaluation and treatment.

## Methods

**Figure 1:**
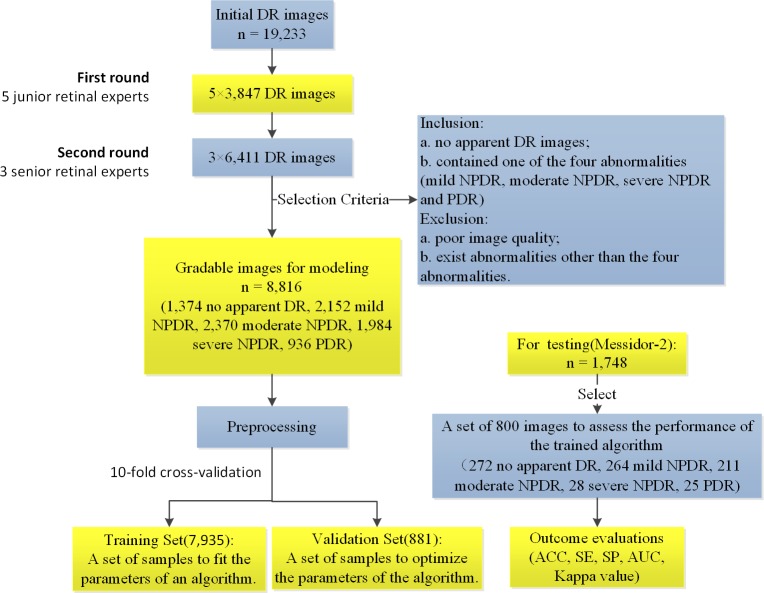
The overall workflow diagram illustrating the main sequential steps for categorizing fundus images in the proposed approach.

The analysis of the proposed deep transfer learning approach with the Inception-v3 network was performed to classify retinal fundus photographs as no apparent DR, mild NPDR, moderate NPDR, severe NPDR, and PDR. The flowchart of the main sequential steps for categorizing DR images in the proposed approach is displayed in Figure 1. First, DR images were obtained, graded, and labeled. The proper image preprocessing technology then was applied to acquire images for better training for the Inception-v3 network using deep transfer learning. After image preprocessing, with the aim of assessing and optimizing our model, we randomly separated the developed image dataset into multiple folds and conducted a 10-fold cross validation. The resulting dataset within each fold was split into two independent training and validation datasets in a 9:1 ratio. Our model parameters were fitted and optimized on the corresponding training dataset and validation dataset, respectively. The performance of our model was investigated using the independent test dataset consisting of 800 images (272 no apparent DR; 264 mild NPDR; 211 moderate NPDR; 28 severe NPDR; 25 PDR) selected from the publically available Messidor-2 dataset. The proposed approach automatically extracted features from many example images with ground truth labels and adjusted its hyperparameters so that the best classification accuracy could be achieved. The identification outcome could assist ophthalmologists in determining whether referral is required. In DR screening, a small number of microaneurysms located in a retinal safe area could be regarded as mild NPDR and would not need referral. However, any patient with moderate NPDR, severe NPDR, or PDR was considered suitable for the referral.

### Dataset

This work was carried out in a compliance with the tenets of the Declaration of Helsinki. In addition, it adhered to the regulation of local ethics committees. The requirement of informed consent was waived as a result of the retrospective nature of this work. A total of 19,233 completely anonymous eye fundus color numerical images were retrospectively collected from a cohort of 5278 adult patients with all stages of DR from Shanghai Zhongshan Hospital and Shanghai First People's Hospital between April 2013 and October 2018. These fundus photographs were taken by multiple color cameras and stored as JPEG files at different resolutions ranging from 1396 × 1396 to 3168 × 4752.

**Figure 2:**
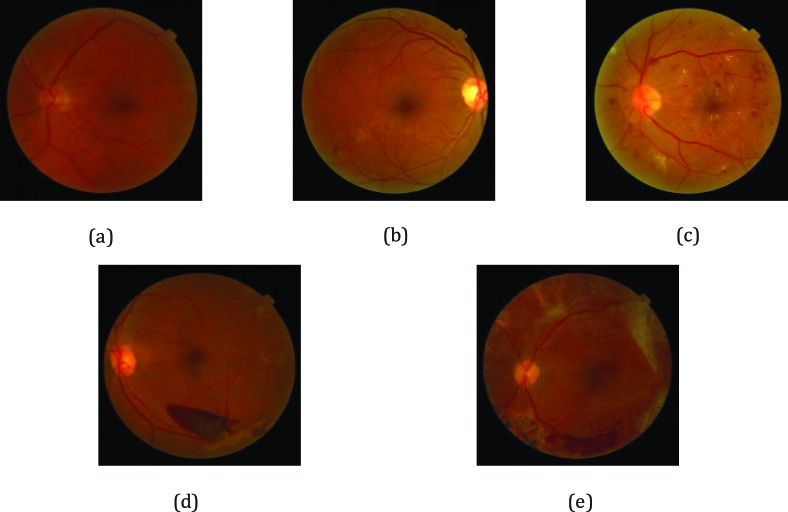
Representation of DR with increasing severity: (a) no apparent DR image; (b) mild NPDR image; (c) moderate NPDR image; (d) severe NPDR image; (e) PDR image.

According to the existence and severity of the diverse DR lesions in the fundus images, the gold standard annotation, namely image labeling, was implemented by retinal experts on the basis of the International Clinical Diabetic Retinopathy severity scale.^20^ The current study invited eight ophthalmologists to grade 19,233 DR images. All images were reviewed masking other clinical information, and each ophthalmologist made a diagnosis independent of other ophthalmologists. In the first round, we randomly assigned these images to five junior retinal experts with 5 to 10 years of experience for screening and labeling. Each expert reviewed about 3847 DR images. During the second review, we invited three senior retinal specialists with 10 to 15 years of experience to verify and rectify the labeling results. Each expert reviewed about 6411 DR images. In the case of discrepancy between retinal experts regarding image labels, labels were adjudicated by an expert committee composed of three senior ophthalmologists, each of whom had more than 20 years of clinical experience. The grading process was carried out using high-resolution 27-inch full-screen monitors. Fundus photographs contained only one of the four abnormalities (mild NPDR, moderate NPDR, severe NPDR, and PDR), and no apparent DR images were included. All images meeting the following criteria were excluded: (1) poor quality image or (2) existence of abnormalities other than the four abnormalities studied. Each fundus photograph was assigned with a diagnostic label of 0, 1, 2, 3, or 4, standing for no apparent DR, mild NPDR, moderate NPDR, severe NPDR, and PDR, as shown in Figure 2. Images with the presence of moderate NPDR or higher were considered as a “refer,” whereas a “no refer” was defined as images with no apparent signs of DR or mild NPDR without clinically significant macular edema (CSME).^21^,^22^

Eventually, the fundus image dataset for the experiment included 8816 images from 5278 adult patients, of whom 1374 were diagnosed as having no apparent DR, 2152 were affected by mild NPDR, 2370 were moderate NPDR patients, 1984 were severe NPDR patients, and the others were PDR patients. For testing, 800 images (272 no apparent DR, 264 mild NPDR, 211 moderate NPDR, 28 severe NPDR, 25 PDR) selected from the publicly available Messidor-2 dataset were used to assess the performance of the trained algorithm. Additionally, we also performed four constituent binary classifiers in the corresponding datasets to distinguish mild NPDR/moderate NPDR/severe NPDR/PDR from no apparent DR to verify the breakdown ability of our model. Moreover, for the purpose of comparing the performance of a deep transfer learning approach on a limited dataset to results obtained on a large dataset, a limited model was also trained to discriminate the same five classifications. During training, 4000 images consisting of 800 images from each category were selected as limited training and validation datasets, while the same test dataset was applied to evaluate the classification performance of the limited model.

### Preprocessing

**Figure 3:**
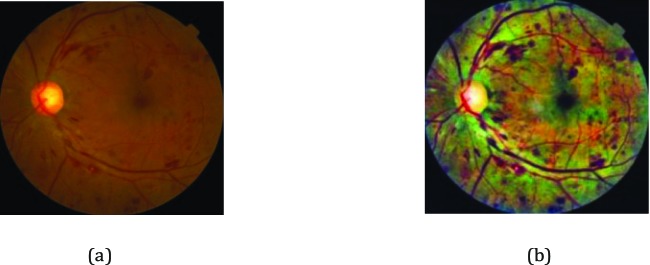
Example of the preprocessing of fundus images: (a) original fundus photograph; (b) fundus photograph after CLAHE application.

Prior to training the deep learning network, multiple preprocessing steps for original images within our dataset were implemented. First, the image pixel values were scaled to range from 0 to 1, and all images were downsized to a uniform 299 × 299 pixel size through properly cropping the inner retinal circle and padding it to a square. In this way, the environmental artifacts in the images that were not related to the diagnosis of DR were also eliminated. Then, considering various image illumination and contrast in retinal images from mass screening, the contrast-limited adaptive histogram equalization (CLAHE) approach^23^ was utilized to enhance the contrast between four DR pathologic signs and background, as shown in Figure 3. Finally, we employed nonlocal means denoising^24^ to remove image noise. The resulting images were presented as the input of Inception-v3 network.

### Deep Transfer Learning Based on Inception-v3 Network

In this study, we selected the Inception-v3 network as the base CNN. This architecture consisted of five convolutional (conv) layers, two max-pooling layers, 11 inception modules, one average pooling layer and one fully connected (fc) layer, which produced an image-wise categorization. The Inception-v3 network clustered the similar sparse nodes into a dense structure to increase both the depth and width of the network and reduce the computation process efficiently. Such a network from scratch had been trained on the ImageNet dataset^25^ with the known ground truth labels, which could identify 1000 categories. In the training process, the pixel intensities of each image and the associated known label were fed into this network so that the parameters in the network were automatically adjusted to make a more accurate prediction based on the performance error defined by the difference between the generated output of the model and the ground truth labels. For each sample image, this process could be repeated multiple times until the network was optimally trained.

**Figure 4:**
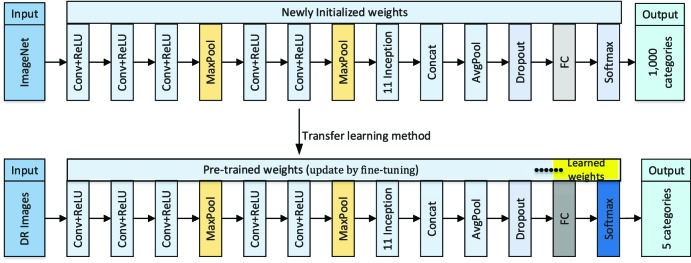
Schematic of deep transfer learning approach with Inception-v3 network.

Considering a relatively small amount of retinal images within our dataset in this study, we transferred the Inception-v3 network, which was pretrained using the ImageNet dataset, and further fine-tuned it for categorization of retinal fundus images in order to reduce training time and to achieve high accuracy. The corresponding schematic of the proposed approach is illustrated in Figure 4. Namely, a deep transfer learning approach with Inception-v3 network was utilized for classifying retinal fundus images. First, we initialized the convolutional layers with the pretrained weights, which were initially calculated from the ImageNet dataset and stored to accelerate training process and reduce redundancy. The last fully connected layers in the Inception-v3 network were initialized with random weights so that the discriminative feature space shifts from ImageNet images to retinal fundus photographs could be learned. Meanwhile, we altered the last fully connected layer to five output categories corresponding to no apparent DR, mild NPDR, moderate NPDR, severe NPDR, and PDR, instead of the 1000 output categories of the ImageNet. Then, all weight parameters of the convolutional layers and corresponding max-pooling layers were frozen to extract features relevant to identification of DR, which meant that the fixed weights would not be changed while the randomly initialized weights would be updated during training. On the development fundus image dataset, each training and validation image was passed through feature extractors, and the resulting output values were used for retraining the newly initialized network to detect our specific five categories. During retraining, an attempt was made to further fine-tune the network by unfreezing frozen layers and updating the corresponding pretrained weights on the development DR image dataset using a back propagation approach until the performance of the validation dataset could not be further improved.

### Statistical Analysis of Performance

For the purpose of evaluating the performance of the proposed deep transfer learning approach for identifying DR, overall classification accuracy (Acc), classification sensitivity (Sen), and specificity (Spe), κ value, and area under the curve (AUC) metrics with 95% CI in the test dataset were calculated, and the performance of the best model was further compared to results obtained by retinal specialists. The statistical analysis was implemented using statistical software (Python ver. 2.7.9) integrating numpy, scipy, statsmodels, and scikit.learn modules. Acc was defined as the ratio of the number of correctly labeled images to the overall number of test images. Sen was calculated by dividing the number of true positive (correctly detected referral cases) by the total number of true positive and false negatives (referral cases incorrectly identified as no referral cases), which showed the percentage of referral cases correctly classified by the algorithm. Spe was defined as a ratio of the number of true negatives (correctly detected no referral cases) to the sum of the number of true negatives and false positives (no referral cases incorrectly identified as referral cases), describing the ratio of no referral cases correctly categorized by the algorithm. Receiver operating characteristic (ROC) curves plotted with respect to the varying operating threshold were used to assess the ability of our model on DR images in discriminating referral from no referral. It provided the tradeoff between the sensitivity and 1-specificity. AUC was utilized for summarizing the classification accuracy of the model. The AUC of effective model ranging between 0.5 and 1 was higher, the performance of model was better. We also calculated a κ value to quantify the degree of agreement between the best performance model and three retinal specialists for each diagnostic category. The larger κ value ranging from 0 to 1 meant better reliability. The Sen, Spe, and Acc were calculated, respectively, as follows:

\newcommand{\bialpha}{\boldsymbol{\alpha}}\newcommand{\bibeta}{\boldsymbol{\beta}}\newcommand{\bigamma}{\boldsymbol{\gamma}}\newcommand{\bidelta}{\boldsymbol{\delta}}\newcommand{\bivarepsilon}{\boldsymbol{\varepsilon}}\newcommand{\bizeta}{\boldsymbol{\zeta}}\newcommand{\bieta}{\boldsymbol{\eta}}\newcommand{\bitheta}{\boldsymbol{\theta}}\newcommand{\biiota}{\boldsymbol{\iota}}\newcommand{\bikappa}{\boldsymbol{\kappa}}\newcommand{\bilambda}{\boldsymbol{\lambda}}\newcommand{\bimu}{\boldsymbol{\mu}}\newcommand{\binu}{\boldsymbol{\nu}}\newcommand{\bixi}{\boldsymbol{\xi}}\newcommand{\biomicron}{\boldsymbol{\micron}}\newcommand{\bipi}{\boldsymbol{\pi}}\newcommand{\birho}{\boldsymbol{\rho}}\newcommand{\bisigma}{\boldsymbol{\sigma}}\newcommand{\bitau}{\boldsymbol{\tau}}\newcommand{\biupsilon}{\boldsymbol{\upsilon}}\newcommand{\biphi}{\boldsymbol{\phi}}\newcommand{\bichi}{\boldsymbol{\chi}}\newcommand{\bipsi}{\boldsymbol{\psi}}\newcommand{\biomega}{\boldsymbol{\omega}}\begin{equation}\tag{1}{\rm{Sen}} = {{TP} \over {TP + FN}}\end{equation}

\newcommand{\bialpha}{\boldsymbol{\alpha}}\newcommand{\bibeta}{\boldsymbol{\beta}}\newcommand{\bigamma}{\boldsymbol{\gamma}}\newcommand{\bidelta}{\boldsymbol{\delta}}\newcommand{\bivarepsilon}{\boldsymbol{\varepsilon}}\newcommand{\bizeta}{\boldsymbol{\zeta}}\newcommand{\bieta}{\boldsymbol{\eta}}\newcommand{\bitheta}{\boldsymbol{\theta}}\newcommand{\biiota}{\boldsymbol{\iota}}\newcommand{\bikappa}{\boldsymbol{\kappa}}\newcommand{\bilambda}{\boldsymbol{\lambda}}\newcommand{\bimu}{\boldsymbol{\mu}}\newcommand{\binu}{\boldsymbol{\nu}}\newcommand{\bixi}{\boldsymbol{\xi}}\newcommand{\biomicron}{\boldsymbol{\micron}}\newcommand{\bipi}{\boldsymbol{\pi}}\newcommand{\birho}{\boldsymbol{\rho}}\newcommand{\bisigma}{\boldsymbol{\sigma}}\newcommand{\bitau}{\boldsymbol{\tau}}\newcommand{\biupsilon}{\boldsymbol{\upsilon}}\newcommand{\biphi}{\boldsymbol{\phi}}\newcommand{\bichi}{\boldsymbol{\chi}}\newcommand{\bipsi}{\boldsymbol{\psi}}\newcommand{\biomega}{\boldsymbol{\omega}}\begin{equation}\tag{2}{\rm{Spe}} = {{TN} \over {TN + FP}}\end{equation}

\newcommand{\bialpha}{\boldsymbol{\alpha}}\newcommand{\bibeta}{\boldsymbol{\beta}}\newcommand{\bigamma}{\boldsymbol{\gamma}}\newcommand{\bidelta}{\boldsymbol{\delta}}\newcommand{\bivarepsilon}{\boldsymbol{\varepsilon}}\newcommand{\bizeta}{\boldsymbol{\zeta}}\newcommand{\bieta}{\boldsymbol{\eta}}\newcommand{\bitheta}{\boldsymbol{\theta}}\newcommand{\biiota}{\boldsymbol{\iota}}\newcommand{\bikappa}{\boldsymbol{\kappa}}\newcommand{\bilambda}{\boldsymbol{\lambda}}\newcommand{\bimu}{\boldsymbol{\mu}}\newcommand{\binu}{\boldsymbol{\nu}}\newcommand{\bixi}{\boldsymbol{\xi}}\newcommand{\biomicron}{\boldsymbol{\micron}}\newcommand{\bipi}{\boldsymbol{\pi}}\newcommand{\birho}{\boldsymbol{\rho}}\newcommand{\bisigma}{\boldsymbol{\sigma}}\newcommand{\bitau}{\boldsymbol{\tau}}\newcommand{\biupsilon}{\boldsymbol{\upsilon}}\newcommand{\biphi}{\boldsymbol{\phi}}\newcommand{\bichi}{\boldsymbol{\chi}}\newcommand{\bipsi}{\boldsymbol{\psi}}\newcommand{\biomega}{\boldsymbol{\omega}}\begin{equation}\tag{3}{\rm{Acc}} = {{TP + TN} \over {TP + TN + FP + FN}}\end{equation}

where, *TP* (true positive) is defined as the overall number of correctly detected referral; *FP* (false positive) is the total number of no referral detected as referral; *FN* (false negative) denotes the overall number of referral detected as no referral; and *TN* (true negative) is the total number of correctly detected no referral.

## Results

**Figure 5:**
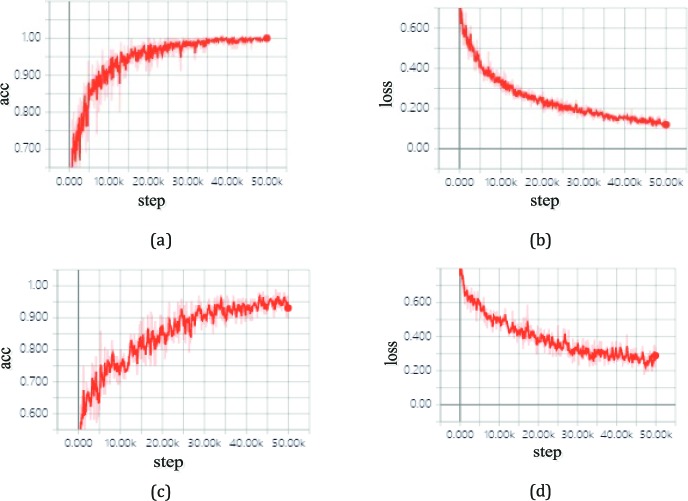
Accuracy and loss on the training and validation datasets with TensorBoard: (a) accuracy versus the training steps; (b) cross entropy loss against the training steps; (c) accuracy versus the validation steps; (d) cross entropy loss against the validation steps.

The proposed deep transfer learning approach with the Inception v3 network was trained and tested on a personal computer (PC) with an Ubuntu 16.04 operation system. The hardware configuration of the PC included Intel Core i7-2700K 4.6-GHz CPU (Intel Corp., Santa Clara, CA), NVIDIA GTX 1080 8-Gb GPU (Santa Clara, CA), Dual AMD Filepro 512-GB PCIe-based flash storage (AMD Corp, Sunnyvale, CA), and 32-GB RAM. For training, the SGD optimizer was utilized with a momentum of 0.95, an initial learning rate of 0.001, weight decay of 0.0005, a batch size of 100 images, and the categorical cross entropy loss function. After 50,000 steps, training on all classifications was stopped as a result of the absence of further improvement in both accuracy and cross entropy loss from then on, as seen in Figure 5.

We measured the time consumed for our model with the configuration previously described. The five-class holdout model trained with our approach spent only about 2 hours in finishing fine-tuning the corresponding datasets, while each binary classification could yield a high accuracy in under 1 hour. Once the model was fine-tuned, 16 milliseconds were needed to assign a DR probability for each retinal image.

**Figure 6:**
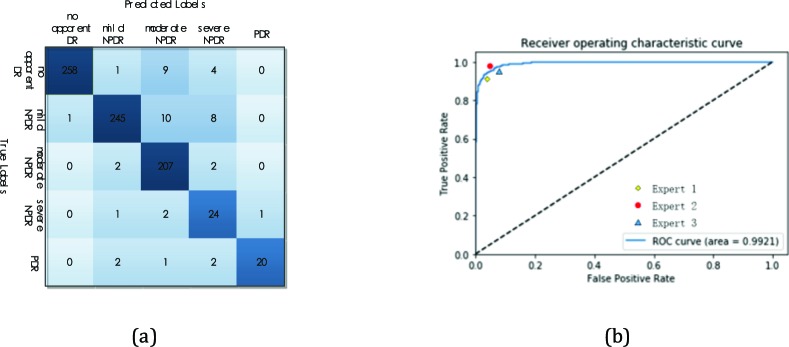
Multiclass comparison between no apparent DR, mild NPDR, moderate NPDR, severe NPDR, and PDR.

The algorithm performance in triaging images with multiclass DR (no apparent DR, mild NPDR, moderate NPDR, severe NPDR, and PDR) was evaluated using the independent Messidor-2 test dataset. The distribution of images over the referral and no referral groups at the stage of training, validation, and testing are in Table 1. Table 2 presents the performance metrics of our model for detecting referral cases. For discrimination between no referral (no apparent DR, mild NPDR) and referral (moderate NPDR, severe NPDR, and PDR), our model obtained an accuracy of 93.49% (95% CI, 93.13%–93.85%) with a sensitivity of 96.93% (95% CI, 96.35%–97.51%), and a specificity of 93.45% (95% CI, 93.12%–93.79%). A ROC curve was generated to evaluate its ability to discriminate no referrals from referrals. The AUC was 0.9905 (95% CI, 0.9887–0.9923). In addition, we further made a performance comparison between the best model and three experts. The best model generated an accuracy of 94.25%, with a sensitivity of 98.11% and a specificity of 94.22%. A confusion matrix of the best model is shown in Figure 6(a). Expert 1 had 93.38% accuracy, 96.21% sensitivity, and 94.59% specificity, while expert 2 and expert 3 had accuracies of 95.13% and 93.87%, a sensitivity of 98.48% and 97.35%, and a specificity of 94.41% and 93.84%, respectively (see Table 3). The κ value for the best model was 0.919, slightly higher than those for the three experts (0.906, 0.931, and 0.914). The ROC curve for referral case identification with human expert performance was plotted to make a comparison as displayed in Figure 6(b). The ROC curve differentiating referral cases from no referral cases had an AUC of 0.9921. It was demonstrated from Figure 6(b) that our best model achieved a performance matching that of human experts.

**Table 1:** Distribution of DR Images Over the Referral and No Referral Groups

Number of DR Images	Referral Group			No Referral Group		Total
	Moderate NPDR	Severe NPDR	PDR	No Apparent DR	Mild NPDR	
Training dataset	2133	1786	842	1237	1937	7935
Validation dataset	237	198	94	137	215	881
Test dataset	211	28	25	272	264	800

**Table 2:** Performance Metrics of Model for Detection of Referrals

Acc, % (95% CI)	Sen, % (95% CI)	Spe, % (95% CI)	AUC (95% CI)
93.49 (0.9313–0.9385)	96.93 (0.9635–0.9751)	93.45 (0.9312–0.9379)	0.9905 (0.9887–0.9923)

**Table 3:** Performance of the Best Model and Human Experts for Detection of the Four Abnormalities

Method	Acc, %	Sen, %	Spe, %	κ
The best model	94.25	98.11	94.22	0.919
Expert 1	93.38	96.21	94.59	0.906
Expert 2	95.13	98.48	94.41	0.931
Expert 3	93.87	97.35	93.84	0.914

**Figure 7:**
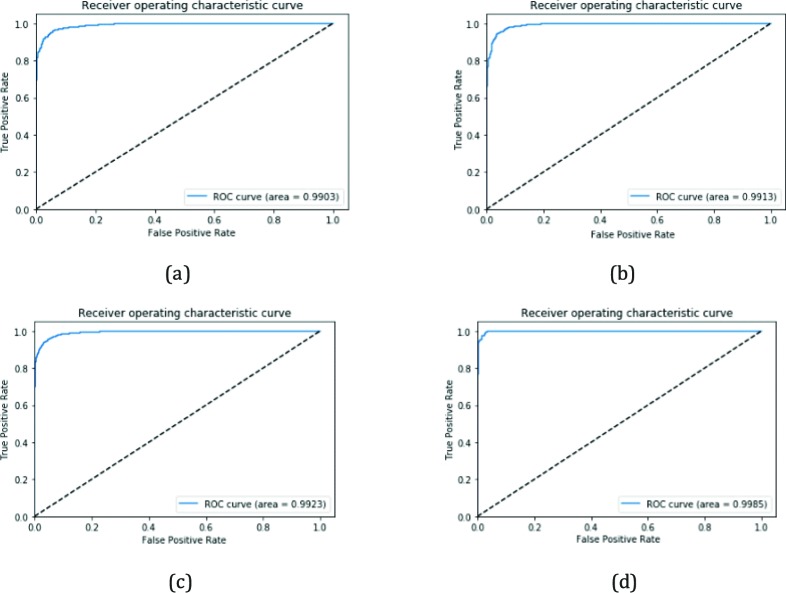
ROC curves of the best four binary classifiers: (a) mild NPDR versus no apparent DR; (b) moderate NPDR versus no apparent DR; (c) severe NPDR versus no apparent DR; (d) PDR versus no apparent DR.

In the following, we performed four constituent binary classifications in detecting the corresponding retinal fundus images to determine a breakdown ability of our model using the corresponding test dataset. Additional analyses were conducted for several subcategories: (1) distinguishing mild NPDR from no apparent DR; (2) discriminating moderate NPDR from no apparent DR; (3) detecting severe NPDR and no apparent DR; (4) separating PDR from no apparent DR. Table 4 lists a summary of these results, which showed the algorithm yielded high accuracy, sensitivity, and specificity. The classifier discriminating mild NPDR from no apparent DR achieved 95.15% (95% CI, 0.9479–0.9551) accuracy, 97.72% (95% CI, 0.9714–0.9827) sensitivity, 92.65% (95% CI, 0.9232–0.9298) specificity, and an AUC of 0.9893 (95% CI, 0.9885–0.9902). The classifier distinguishing moderate NPDR from no apparent DR yielded an accuracy of 94.41% (95% CI, 0.9407–0.9475) with a 97.64% (95% CI, 0.9706–0.9822) sensitivity and 91.91% (95% CI, 0.9161–0.9221) specificity, and an AUC of 0.9904 (95% CI, 0.9898–0.9910). The classifier differentiating between severe NPDR and no apparent DR obtained an accuracy of 92.67% (95% CI, 0.9236–0.9298), a 97.43% (95% CI, 0.9688–0.9798) sensitivity, and a 92.28% (95% CI, 0.9196–0.9260) specificity, while the value of AUC reached up to 0.9912 (95% CI, 0.9905–0.9918). The classifier separating PDR from no apparent DR achieved an accuracy of 98.65% (95% CI, 0.9835–0.9895), with a 99.26% (95% CI, 0.9873–0.9979) sensitivity and 98.53% (95% CI, 0.9800–0.9884) specificity, while the AUC score was 0.9977 (95% CI, 0.9971–0.9983). The ROC curves for discriminating referral from no referral with the best four binary classifiers were depicted in Figure 7.

**Table 4:** Performance Comparisons of Our Model for Binary Classification

Method	Acc, % (95% CI)	Sen, % (95% CI)	Spe, % (95% CI)	AUC (95% CI)
Mild NPDR vs no apparent DR	95.15 (0.9479–0.9551)	97.72 (0.9714–0.9827)	92.65 (0.9232–0.9298)	0.9893 (0.9885–0.9902)
Moderate NPDR vs no apparent DR	94.41 (0.9407–0.9475)	97.64 (0.9706–0.9822)	91.91 (0.9161–0.9221)	0.9904 (0.9898–0.9910)
Severe NPDR vs no apparent DR	92.67 (0.9236–0.9298)	97.43 (0.9688–0.9798)	92.28 (0.9196–0.9260)	0.9912 (0.9905–0.9918)
PDR vs no apparent DR	98.65 (0.9835–0.9895)	99.26 (0.9873–0.9979)	98.53 (0.9800–0.9884)	0.9977 (0.9971–0.9983)

In the following, the limited model was also further trained based on a transfer learning approach on limited data in order to compare with results obtained using a large dataset. We selected a total of only 4000 images (800 images extracted randomly from each category) as a limited training dataset to train our model to identify the same five categories. On the same test dataset, the limited model achieved a relatively good performance of 93.16% (95% CI, 0.9275–0.9357) accuracy with a sensitivity of 96.75% (95% CI, 0.9613–0.9737) and specificity of 93.18% (95% CI, 0.9276–0.9360), while the AUC was 0.9864 (95% CI, 0.9846–0.9886), which is shown in Table 5. This illustrated that the proposed approach had great performance and could detect DR automatically and accurately.

**Table 5:** Performance Metrics of Limited Model for Detection of Referrals

Acc, % (95% CI)	Sen, % (95% CI)	Spe, % (95% CI)	AUC (95% CI)
93.16 (0.9275–0.9357)	96.75 (0.9613–0.9737)	93.18 (0.9276–0.9360)	0.9864 (0.9846–0.9886)

## Discussion

In the present work, a deep transfer learning approach with Inception-v3 network was presented to identify the level of DR from retinal fundus photographs automatically that achieved high accuracy, sensitivity, and specificity. This approach avoided a great deal of example images for convergence of the model via fine-tuning the weights of the Inception-v3 network, which was pretrained using the ImageNet dataset and yielded a matching or exceeding performance to that of retinal specialists in detecting DR images.^26^,^27^ The results attained indicated that our approach could provide better consistent predictions and highly reliable detection without having to specify lesion-based features, and it could serve as an automated screening tool for early DR by using retinal fundus images in addition to assisting ophthalmologists in making a referral decision.

Retinal fundus image interpretation is often subjective and liable to significant inter- and intraobserver variability, even among experienced ophthalmologists. Considering these limitations, automated DR detection methods would be of enormous value. The proposed approach for DR detection offered consistency of interpretation on a specific image. The performance of the proposed approach was yielded directly by the results of the training data, with a human expert grading decisions, without the need to focus on underlying process of DR. In addition, when performing a large-scale screening for DR, it was critical to improve sensitivity and specificity for minimizing misdiagnosed cases. Our approach offered good results in the sensitivity and specificity, while a near instantaneous reporting ability of results could also be achieved. In this study, 93.49% (95% CI, 93.13%–93.85%) accuracy, 96.93% (95% CI, 96.35%–97.51%) sensitivity, and 93.45% (95% CI, 93.12%–93.79%) specificity were generated, while the AUC was up to 0.9905 (95% CI, 0.9887–0.9923), manifesting a comparable or slightly better performance than the previous studies.^17^,^18^,^28^ Moreover, the most significant merit of our approach was possibly the endeavor to simultaneously predict five levels of DR with improved performance compared to previous studies where only four DR grades were considered,^29^ which was suitable for more timely and reliable detection of DR.

Most artificial intelligence studies using retinal fundus images concentrated on explicit handcrafted feature engineering involving computing and extracting complex features,^30^ which was time-consuming, required considerable skill and professional knowledge for annotating the imaging data, and easily resulted in misclassification due to a minor error in handcrafted engineering features. However, the key advantage of our approach was that it could learn automatically richer and more distinctive image features from the retinal fundus image data to achieve more accurate identification, without manual feature extraction or feature optimization. This autonomous behavior could present a potential opportunity for capturing subtle characteristics or patterns of DR in clinical settings, which may not be identified by retinal experts. Additionally, the approach developed in this study did not require any specialized or advanced computer equipment to classify fundus photographs, and it could be deployed on standard low-cost computing equipment to offer reproducible evaluation of DR images in patients with suspected DR diseases.

In our system, we transferred the pretrained Inception-v3 model and adjusted the last fully connected layer to five output categories exactly corresponding to our multiclass identification task instead of the 1000 output categories of the ImageNet. Subsequently, weights of the pretrained Inception-v3 model were placed into the transferred model, while weight parameters in the last fully connected layer were randomly initialized. During the fine-tuning, the strategies for the hyperparameter setting and searching were different from that in the training process. First, the initial learning rate was set to much less than 0.1 through the optimization algorithm for training the model well during the fine-tuning. Moreover, there was no need to update all weight parameters of the model due to limited training data. The most effective way to fine-tune the pretrained weight parameters of the CNN model was to adjust only those parameters in the fully connected layer most relevant to specific fundus photograph classification, while fixing the weight parameters of convolutional layers and the corresponding pooling layers. In our system, the fine-tuning process was performed for 50,000 steps using the SGD optimizer with a batch size of 100. The learning rate was initially set to 0.001 and was then decreased linearly from 0.001 to 0.0001 over 150 epochs in the training process. The categorical cross entropy loss function was utilized, whereas the values of weight decay and momentum were set to 0.0005 and 0.95, respectively. During retraining, the frozen layers were attempted to further fine-tune through unfreezing and adjusting the corresponding pretrained weight parameters on the developed DR photograph dataset using a back propagation approach until the performance of the validation dataset could not be further improved. Once the optimal learned weights were determined, the work procedure of our system was in according with that of the conventional CNN.

Limitations of transfer learning approach must be considered. First, although the transfer learning approach achieved satisfactory results in detection of DR and decreased the training time for model convergence on a relatively small training dataset, it exhibited slightly inferior classification power in contrast to that of the model trained from scratch on a huge training dataset. The reason was mainly due to the fact that the weight parameters of the model trained from scratch could be directly updated and optimized for DR feature identification. Second, when source and target domains have little relevance to each other in some sense, transfer learning may lead to a decline of performance. Also, the performance of the developed model was determined by weight parameters of the pretrained Inception-v3 model to a large extent, which could be further improved by testing with a larger ImageNet dataset. Also, when an attempt to fine-tune the network via unfreezing and updating pretrained weight parameters on the developed DR image dataset with the back propagation method, overfitting was prone to occur, resulting in a decline of model performance. However, transfer learning accelerated the training of the model, reduced memory complexity, and yielded a high classification accuracy between no apparent DR, mild NPDR, moderate NPDR, severe NPDR, and PDR on a relatively small DR photograph dataset. The deep transfer learning approach with Inception-v3 network could accurately capture features of DR images; as a result the relative high performance in automated DR identification could be generated. Unfortunately, it could be extremely expensive or unfeasible to collect a large amount of DR images as the underlying datasets with the gold standard qualified by ophthalmologists. However, even if they could be collected, the training of a deep CNN would also require extensive memory, computational resources, and several weeks to update the substantial hyperparameters of the model to converge to a high accuracy. In contrast, a multiclass holdout model trained through the use of a deep transfer learning approach could save memory, reduce computational resources, and only take approximately 2 hours to finish training, validating, and testing in the corresponding datasets. We also trained four constituent binary classifications identifying mild NPDR/moderate NPDR/severe NPDR/PDR from no apparent DR and the limited model independently. Each binary classification and the limited model showed excellent performance and could also generate a relatively high accuracy in about 1 hour. Thus, the initializations of models with the deep transfer learning approach should be regarded as a critical method when a CNN was trained for performing a new task, especially with limited data.

Nevertheless, there also exists several limitations to our approach in the current study. First, we selected the retinal fundus images from only two hospitals. Various device settings, camera systems, and population characteristics had impacts on DR images and further affected the model's performance. In order to further evaluate our approach, we need to collect more retinal fundus image data from different hospitals and use larger patient cohorts in future studies. Second, since the deep learning model was referred to as black boxes, it was difficult to know how the algorithm analyzes features and makes predictions for DR images. In particular, when difficult and ambiguous cases occurred, it was very useful to make an objective interpretation. Thereby, the visualization of the decision-making process of the model needs to be studied further. This raised the possibility that visualizing model decisions could potentially aid both patients and physicians in real-time clinical verification. Third, our approach learned the features based only on the fundus images and their associated grades, rather than explicit, defined features. Therefore, it is possible that the algorithm was using some features ignored by humans to predict classification results. In subsequent studies, we need to gain insight into how the deeply neural network analyzes patterns and makes an image-wise prediction.

## Conclusions

In our present study, a novel deep transfer learning approach with the Inception-v3 network was developed to automatically detect DR in retinal fundus images. The proposed approach had high accuracy, high sensitivity, and high specificity for classifying DR images; offered consistency of interpretation on the specific DR image; and could assist ophthalmologists in making a referral decision. Moreover, this approach did not need to extract engineered features from the retinal fundus photograph dataset for DR detection. In addition, the process of DR detection in the proposed approach was fully autonomous. Thus, it could serve as an important role in making automated screening for early DR based on retinal fundus photographs. In future studies, it will be necessary to determine the decision-making process of the model and validate the generalization of our approach. Additionally, in order to extend the deep transfer learning approach with the Inception-v3 network to real clinical applications, there is need to turn the proposed method into applied software so that it could act as a DR screening tool and provide decision-making support for ophthalmologists.
